# Glycan Shielding and Modulation of Hepatitis C Virus Neutralizing Antibodies

**DOI:** 10.3389/fimmu.2018.00910

**Published:** 2018-04-27

**Authors:** Muriel Lavie, Xavier Hanoulle, Jean Dubuisson

**Affiliations:** ^1^University of Lille, CNRS, INSERM, CHU Lille, Institut Pasteur de Lille, U1019 – UMR 8204 – CIIL – Center for Infection & Immunity of Lille, Lille, France; ^2^University of Lille, CNRS, UMR 8576, UGSF, Unité de Glycobiologie Structurale et Fonctionnelle, Lille, France

**Keywords:** hepatitis C virus, neutralizing antibodies, glycosylation, humoral immune response, glycoproteins

## Abstract

Hepatitis C virus (HCV) envelope glycoprotein heterodimer, E1E2, plays an essential role in virus entry and assembly. Furthermore, due to their exposure at the surface of the virion, these proteins are the major targets of anti-HCV neutralizing antibodies. Their ectodomain are heavily glycosylated with up to 5 sites on E1 and up to 11 sites on E2 modified by N-linked glycans. Thus, one-third of the molecular mass of E1E2 heterodimer corresponds to glycans. Despite the high sequence variability of E1 and E2, N-glycosylation sites of these proteins are generally conserved among the seven major HCV genotypes. N-glycans have been shown to be involved in E1E2 folding and modulate different functions of the envelope glycoproteins. Indeed, site-directed mutagenesis studies have shown that specific glycans are needed for virion assembly and infectivity. They can notably affect envelope protein entry functions by modulating their affinity for HCV receptors and their fusion activity. Importantly, glycans have also been shown to play a key role in immune evasion by masking antigenic sites targeted by neutralizing antibodies. It is well known that the high mutational rate of HCV polymerase facilitates the appearance of neutralization resistant mutants, and occurrence of mutations leading to glycan shifting is one of the mechanisms used by this virus to escape host humoral immune response. As a consequence of the importance of the glycan shield for HCV immune evasion, the deletion of N-glycans also leads to an increase in E1E2 immunogenicity and can induce a more potent antibody response against HCV.

## Introduction

With approximately 70 million people chronically infected worldwide, hepatitis C virus (HCV) is a major health burden. In most cases, HCV establishes chronic infection that can lead to the development of cirrhosis and hepatocellular carcinoma. For a long time, standard treatment for HCV infection consisted in a non-specific combination therapy with pegylated interferon and ribavirin, which was relatively toxic and effective in half of treated patients. Advances in *in vitro* and *in vivo* HCV infection systems resulted in a great increase of our understanding of the HCV life cycle. This led to the development of several successful direct acting antivirals that allow for the achievement of high HCV clearance rates (>90%). However, the high cost of these antivirals therapy precludes their accessibility to the large majority of HCV-infected patients (1). In this context, the development of a preventive HCV vaccine would constitute the most cost-effective means to limit HCV spread. Studies have shown that a successful HCV vaccine would induce the production of neutralizing antibodies and a potent HCV-specific T cell response (2). However, a key challenge in HCV vaccine development is to overcome the high diversity of this virus. Several vaccine candidates targeting the envelope glycoproteins have been shown to induce strong humoral and cellular immune response in animal models or clinical trials in humans. However, their efficiency was limited by viral escape from immune response due to the high genetic variability of the virus (2–5). In this context, the design of an efficient vaccine will require a good knowledge of the strategies used by the virus to escape host immune response. One of these strategies is the presence of a glycan shield that protects E2 conserved epitopes from neutralizing antibodies. Here, we present the glycosylation of HCV envelope glycoproteins and we review the different aspects of the modulation of neutralizing antibodies by HCV glycan shield.

## Glycosylation of HCV Envelope Proteins

### Distribution of E1 and E2 N-Glycans

**Figure 1:**
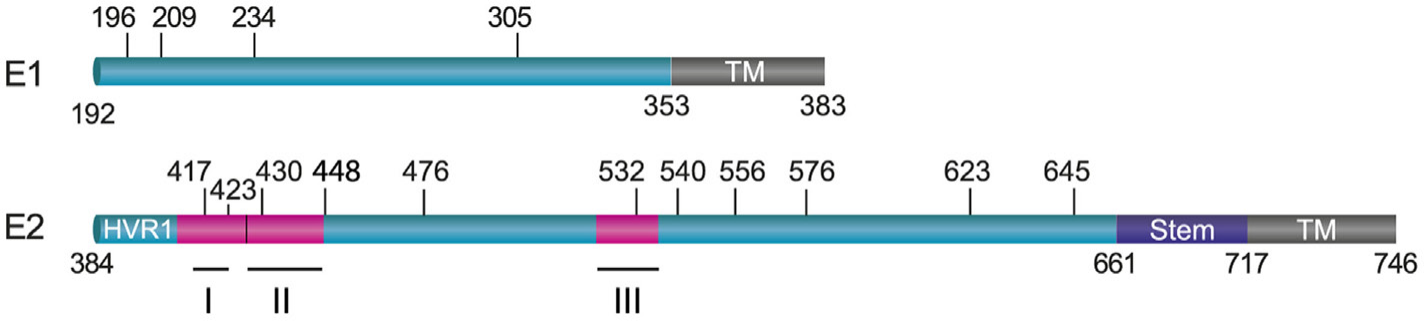
Position of N-linked glycans on hepatitis C virus envelope glycoproteins. E1 and E2 are schematically represented by boxes with their transmembrane domains shown in brown. The glycosylation sites and their position are indicated by vertical bars (on reference strain H77). The localization of three major neutralizing epitopes on E2 (I: 412–423; II: 427–446; III: 523–535) is also shown.

E1 and E2 are highly glycosylated with N-glycans representing one-third of the heterodimer mass. N-glycosylation occurs on the asparagine (Asn) residue belonging to aparagine–X–serine/threonine (Asn–X–Thr/Ser) motifs where X denotes any residue but Proline. In most genotypes, E1 contains four conserved glycosylation sites that are located at amino acid position 196 (E1N1), 209 (E1N2), 234 (E1N3), and 305 (E1N4) in genotype 1a H77 strain (Figure 1). However, an additional glycosylation site is present at position 250 in genotypes 1b and 6, or at position 299 in genotype 2b (6).

Up to 11 glycosylation sites can be detected in most E2 glycoprotein sequences. Nine of them are conserved across HCV genotypes, and they are located at positions 417 (E2N1), 423 (E2N2), 430 (E2N3), 448 (E2N4), 532 (E2N6), 556 (E2N8), 576 (E2N9), 623 (E2N10), and 645 (E2N11) in the H77 reference strain (Figure 1). The two other glycosylation sites are also conserved in most genotypes except in genotype 1b for the site at position 476 (E2N5) and in genotypes 3 and 6 for the site at position 540 (E2N7). Thus, despite high sequence variability in HCV, the majority of N-glycosylation sites are highly conserved, suggesting that glycans play a major role in the HCV life cycle. Importantly, all these sites have been confirmed to be occupied by glycans (7, 8).

In a minority of HCV genomes, additional glycosylation sites can also be observed. For instance, another glycosylation site has been reported to be present in the intragenotypic hypervariable region HVR495 of E2 in a minority of genotype 3a isolates from Pakistani patients (9). An additional glycosylation site has also been shown to appear in hypervariable region 1 (HVR1) after selection of a mutant resistant to monensin treatment in cell culture (10). The appearance of such natural or selected glycans suggests that HCV can adapt to environmental changes by generating novel glycosylation sites.

### Type of Glycans Associated With E1 and E2 Glycoproteins

N-linked glycosylation occurs by the transfer en bloc of a Glc3Man9GlcNAc2 oligosaccharide from a lipid intermediate to an Asn residue in the consensus sequence Asn–X–Thr/Ser of a nascent protein. Three major types of N-glycans can be observed on glycoproteins. The first type corresponds to high mannose glycans which are composed of two *N*-acetylglucosamine molecules linked to several mannoses residues; the second type of glycans are complex oligosaccharides which are mainly composed of two *N*-acetylglucosamines, galactose and can contain sialic acid and fucose. Hybrid type glycans constitute the third type of N-glycans. They are composed of *N*-acetylglucosamine, galactose, mannose, and can contain sialic acid. Complex and hybrid glycans are generated during the transit of the protein through the Golgi compartment by addition or removal of sugar residues by specific enzymes. It has been shown that cell-associated E1E2 mainly display high-mannose-type oligosaccharides, which is in agreement with their retention in the endoplasmic reticulum (ER) of infected cells (11). Furthermore, the characterization of E2 glycosylation sites by mass spectrometry confirmed that the majority of these sites are occupied by high mannose glycans on a recombinant form of the glycoprotein (12).

Since they are assembled in the ER, HCV particles have to cross the secretory pathway before being released by infected cells. During this process, the glycoproteins associated with the virions are modified by cellular glycosidases and glycosyl transferases. In the absence of a cell culture system for HCV, the first evidence of glycan modifications linked to secretion of viral particles came from the characterization of retroviral pseudotypes harboring HCV envelope glycoproteins [HCV pseudoparticles (HCVpp)] (13, 14). However, later on, different patterns of glycosylation have been observed between cell culture-derived HCV (HCVcc) and HCVpp associated glycoproteins (11). Thus, HCVcc-associated E1E2 heterodimers contain both high-mannose and complex type N-linked glycans, whereas HCVpp associated E1E2 display a majority of complex-type glycans. Complex glycans are hallmarks of protein transit through the Golgi apparatus since they result from the processing of high-mannose-type glycans by Golgi glycosidases and glycosyltransferases (15). The incomplete maturation of HCVcc E1E2 glycans indicates that some glycans are not accessible to Golgi enzymes. By contrast, HCVpp-associated glycans are more efficiently matured. These results are likely due to differences in the assembly process of HCVpp and HCVcc. Indeed, HCVcc assemble in an ER-derived compartment (16), whereas HCVpp assemble in a post-Golgi compartment (17). Therefore, in the HCVpp system, E1E2 transit through the Golgi compartment without any other viral components and are thus fully accessible to Golgi enzymes. In the HCVcc system, E1E2 are already associated with nascent viral particles when they travel through the secretory pathway and might thus be less accessible to Golgi enzymes.

It is worth noting that, in addition to N-linked sugars, O-linked glycans have also been identified on a recombinant form of E2 protein (18). Four of these O-linked carbohydrates were identified in HVR1 and two in the core structure of E2 (Thr473 and Thr518). However, these types of glycans were not found by another group which used a similar approach for their detection (19). Since the two groups used an E2 protein from different genotypes, one cannot exclude genotype differences in terms of O-glycosylation, but no O-linked glycans were reported on the structure of E2 (20, 21). It is also possible that the O-glycans observed by Braütigam and coworkers (18) are present on a misfolded structure of E2.

### N-Glycans on E1 and E2 Structures

In 2014, the crystal structure of the N-terminal sequence (residues 192–270) of E1 expressed in the absence of E2 was obtained. This polypeptide contained N-glycosylation sites E1N1 and E1N2, but the E1N3 N-glycosylation site was removed from the sequence by mutagenesis. However, to facilitate the crystallization process, the molecule was produced in the presence of an N-glycosylation inhibitor (22). In this structure, the N-terminus forms a beta-hairpin followed by a domain composed of a 16 amino acid long alpha-helix flanked by a three strands anti-parallel beta-sheet. The oligomeric arrangement displays two types of dimers. In the first type, the two monomers interface is formed by the interaction of the N-terminal beta-hairpin forming an anti-parallel beta-sheet and by hydrogen bonding between Y1 residue and the *N*-acetyl-d-glucosamine of the E1N1 glycosylation site. The second dimer interface corresponds to a six-stranded beta-sheet formed from two sets of three strands from two monomers that is stabilized by two disulfide bridges.

**Figure 2:**
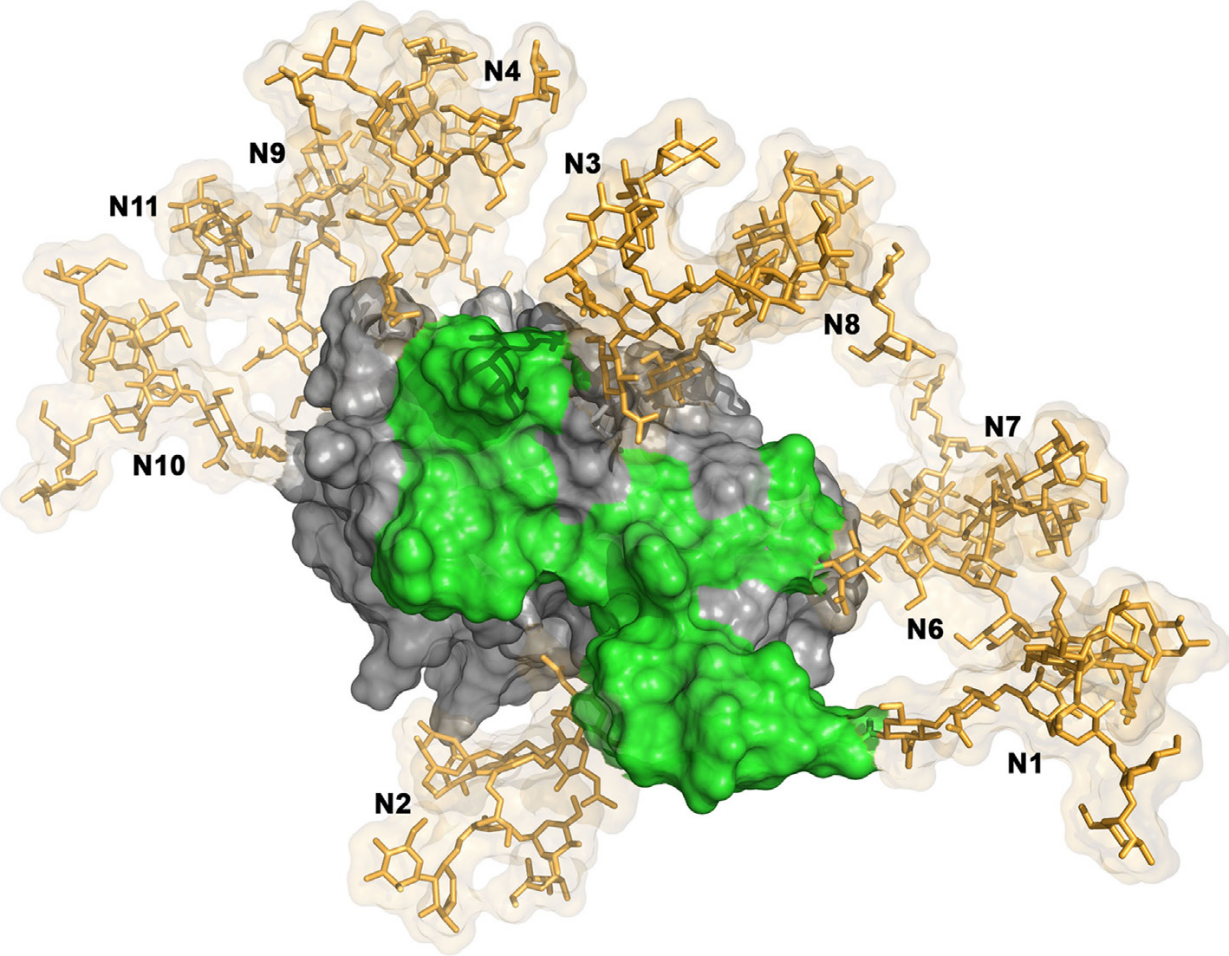
Glycan shield masking E2 neutralizing epitopes. The model of E2 structure is composed by the E2 core structure (PDB ID: 4MWF) (20) and its N-terminal antigenic region 412–423 (PDB ID: 4DGY) (23). The E2 structural model has been built in a similar way than in Fuerst et al. (24). The hypervariable region 1 located at the N-terminus of E2 is not shown. The E2 molecular surface is displayed in gray and its neutralizing epitopes are highlighted in green. High mannose N-glycans (Man9GlcNac2) have been modeled at the 10 N-glycosylation sites available in the E2 structural model using the Glycoprotein Builder tool of the GLYCAM-webserver (http://glycam.org), with an energy minimization step. The glycans (N1, N2, N3, N4, N6, N7, N8, N9, N10, and N11) are shown in sticks representation (in gold) with their transparent molecular surface. The figure was generated with PyMOL (The PyMOL Molecular Graphics System, Version 1.83 Schrödinger, LLC).

The structure of the central E2 ectodomain was solved by two independent groups in 2013 and 2014 (20, 21). The obtained structures were very similar [residues 412–645 of E2 of E2 from H77 isolate of genotype 1a in Kong et al. (20); residues 456–656 of E2 from J6 isolates of genotype 2a in Khan et al. (21)]. E2 core shows a globular structure. Indeed, it is composed of a central immunoglobulin fold beta domain as found in other viral envelope proteins. This central beta sandwich is flanked by front and back layers consisting of loops, short helices, and beta sheets. Most of the N-glycosylation sites present on E2 could be observed in the crystal structure (H77 strain sequence) obtained by Kong and collaborators (4MWF) (20). Only E2N1, E2N5, E2N4, and E2N9 are absent due to truncations or mutations introduced in the E2 sequence to facilitate crystallization (20). The majority of the N-linked glycans were disordered in the crystal structure. Only glycan N430 could be modeled as Man6GlcNAc2. E2 structure revealed that 7 of the 11 N-linked glycans form an extensive glycan shield that masks E2 neutralizing epitopes (Figure 2) (20). Residues E2N7, E2N8, E2N10, and E2N11 were also modeled in the final E2 core structure obtained by Khan and collaborators (21). They were located on the periphery of the core on a highly basic surface.

## Role of E1E2 N-Glycans in the HCV Life Cycle

### Role of the N-Glycans of E1 and E2 in the Early Secretory Pathways

One of the major roles of N-linked glycans is their involvement in protein folding (15). Indeed, the presence of large polar saccharides directly influences the local orientation of the protein. In addition, N-glycans indirectly affect protein folding through their interaction with the ER chaperones, calnexin, and calreticulin. Calnexin and calreticulin are lectin-like chaperones, which show an affinity for monoglucosylated N-linked oligosaccharides (15, 25). Calnexin has been shown to interact with HCV envelope glycoproteins (26–28) and has been suggested to be involved in the folding of E1E2 envelope glycoproteins. In the case of HCV, several N-linked glycans of E1 (E1N1 and E1N4) and E2 (E2N7, E2N8, and E2N10) have been shown to play a role in E1 and E2 folding and heterodimerization. The alteration of the folding observed for these mutants was not due to a lack of recognition by the calnexin chaperone (8).

### Functions of Virion-Associated Glycans

The extended glycosylation of E1 and E2 suggests that interactions between lectin receptors and virus might play a role during HCV infection. In agreement with this hypothesis, several lectin receptors have been shown to participate in HCV entry (29). Thus, HCV is thought to initially bind the endothelium of the liver *via* the mannose-binding lectins L-SIGN and the dendritic cells *via* DC-SIGN. Both cell surface proteins are believed to function as capture receptors that concentrate the virus before subsequent interaction with the hepatocytes (29). HCV interaction with hepatocytes is then mainly promoted by scavenger receptor BI (SR-BI), CD81, and the tight-junction proteins claudin-1 and occludin. Interestingly, the type of glycans associated with E1E2 proteins has been shown to influence their binding affinity for lectin receptors as well as for the non-lectin receptors (30).

Site-directed mutagenesis in HCVpp and HCVcc systems enabled to further characterize the functional role of N-glycans associated with HCV envelope proteins (8, 31, 32) (Table 1). These studies confirmed that several glycans (E1N1, E2N8, and E2N10) are involved in E1E2 folding and heterodimerization. Glycans can also modulate E1E2 entry functions by affecting the affinity of the envelope proteins for receptors. Indeed, mutation of E2N6 glycosylation site led to an increase in HCVcc infectivity. Moreover, this mutant was more sensitive to infectivity inhibition by a soluble form of the CD81 large extracellular loop (32). In agreement with these data, a soluble form of E2 devoid of E2N6 glycan exhibited a higher affinity for CD81 (31). Altogether, these data suggest that the improved infectivity of E2N6 mutant is due to its increased affinity for CD81. Interestingly, the loss of E2N6 glycosylation site has been observed among naturally occurring HCVcc variants adapted to cell culture (33, 34). It is worth noting that on the 3D structure of E2, the CD81-binding site is surrounded by glycans (20), and the removal of the glycan at position E2N6 likely provides more space for CD81 binding.

**Table 1:** Summary of the features of HCV glycosylation mutants [adapted from Helle et al. (92)].

Virus	HCVcc Infectivity^a^	HCVpp infectivity^a^^,^^b^	Core release^c^	Sensitivity to neutralization^d^
wt	+++	+++	++	+
**Mutant**				
E1N1	+/−	++	−	ND (+)
E1N2	++	+	+	ND (+)
E1N3	+++	++	++	ND (+)
E1N4	++	+	+/−	ND (+)
E2N1	+++	++	++	++
E2N2	++	− (−)	++	++^e^
E2N3	+	+++	+	ND (+)
E2N4	++	− (−)	+	++^e^
E2N5	++	++	++	+
E2N6	+++	++	++	++
E2N7	+/−	+++ (+)	+	ND (−)
E2N8	−	−	+/−	ND
E2N9	+++	+++	++	+
E2N10	−	−	−	ND
E2N11	+	+	+/−	++
HVR495	+++	+	ND	++

*^a^Percentage of infectivity relative to the wild type (wt): +++, >90%; ++, between 30 and 90%; +, between 10 and 30%; +/−, between 2 and 10%; −, <2%*.

*^b^Infectivity of HCVpp of genotype 1a and 3a for the HVR495 glycan (9). The results in brackets are obtained for genotype 2a HCVpp*.

*^c^Percentage of core release relative to the wt: ++, >75%; +, between 30 and 75%; +/−, between 12 and 30%; −, <12%*.

*^d^Sensitivity to antibody neutralization: +, similar to the wt; ++, more than fourfold increase in sensitivity to neutralization with most antibodies tested; −, decrease in sensitivity to neutralization. The values in brackets were obtained for genotype 1a HCVpp only*.

*^e^Results obtained with the HCVcc system only*.

*ND, not determined; HCV, hepatitis C virus; HCVpp, HCV pseudoparticles; HCVcc, cell culture-derived HCV*.

By contrast, mutation of the E2N7 glycosylation site led to a strong decrease in HCVcc infectivity without affecting viral particle secretion, suggesting that the glycan present at this position modulates virus entry (32). However, the exact mechanism was not determined. Furthermore, the role of E2N7 glycan in virus entry is likely genotype specific since this glycan site is absent in genotype 3 and 6.

### Different Roles for N-Glycans in HCVpp and HCVcc Systems

Noteworthy, in some cases, envelope glycoprotein entry functions were differently affected by glycan loss in HCVcc and HCVpp systems (32). It is the case for the E2N2 or E2N4 mutations that slightly affected HCVcc infectivity but abolished HCVpp infectivity (32). Several studies could also demonstrate differences between the entry functions of envelope proteins in HCVpp and HCVcc systems (35–39). These differences might be due to the distinct assembly processes of HCVcc and HCVpp particles, that lead to different glycan processing and different organization of the proteins at the virion surface (11, 14, 17, 40). Moreover, the association of HCVcc with lipoproteins might also account for the differences in the properties of E1E2 in HCVpp and HCVcc systems (40, 41). Importantly, despite differences in glycosylation patterns, there is a strong correlation between HCVpp and HCVcc in their sensitivity to antibody neutralization, indicating that the type of glycans associated with HCV envelope proteins might not drastically affect the recognition of neutralizing epitopes (39).

### Antiviral Strategies Targeting E1E2 N-Glycans

The importance of N-glycans for the folding of E1E2 envelope proteins, for HCV entry and for the protection of the virus from neutralizing antibodies make them promising targets for antiviral strategies. Accordingly, many studies have shown the antiviral activity against HCV of several carbohydrates binding agent (CBA), such as cyanovirin-N, griffithsin, or scytovirin lectins, as well as pradimicin-A (42–47). Surprisingly, the selection of resistance mutations by propagating HCVcc in the presence of increasing doses of CBA did not lead to the appearance of mutations in the envelope glycoproteins. Resistance was rather conferred by mutations in the core and the non-structural proteins (48), suggesting indirect mechanisms of resistance.

In agreement with the functional role of glycosylation in the HCV life cycle, inhibition of α-glycosidases I and II, that are essential for N-linked glycan processing, impaired HCV production. Indeed, treatment of infected cells with competitive inhibitors of α-glucosidases led to the degradation of E2 and to the consequent inhibition of HCV assembly and secretion (49, 50). Despite the great potential of glycosidase inhibitors as broad-spectrum antiviral drugs, their clinical development has been hampered by their relatively low efficacy. Such a compound like celgosivir showed only a modest antiviral effect in chronically HCV-infected patients in a phase II clinical trial (5% of the tested patients experienced a 10-fold reduction in viremia) (51).

## HCV Glycan Shield and Host Immune Response

### Neutralizing Determinants in HCV Envelope Glycoproteins

Neutralizing antibodies inhibit viral infection by binding to viral particles. This leads to the blockade of the interaction with receptors or prevents the envelope glycoproteins conformational changes required for the fusion step. E2 envelope glycoprotein is the main target of the humoral immune response against HCV (52). The importance of neutralizing antibodies to eliminate HCV infection has been shown in a humanized mouse model (53). Several regions of E2 are targeted by neutralizing antibodies. Among them the first 27 amino acids of E2 that correspond to HVR1, a highly variable region of the protein, play important roles in interaction with the HCV co-receptor SR-BI, in viral fitness, and in assembly and release of viral particle (54–57). However, antibodies targeting HVR1 exhibit poor cross-neutralization potency across HCV genotypes due to the high variability of this region (58). HVR1 deletion mutants are more susceptible to neutralization by monoclonal antibodies (mAbs) and patient sera (54, 56). Since CD81-binding site is the main target of neutralizing antibodies, this finding suggests that HVR1 masks this site on E2. Additional lines of evidence gave rise to this hypothesis. Indeed, no interaction could be observed between HCV and CD81 at the cell surface in the absence of SR-BI (59). This led to the proposal that HVR1 shields CD81-binding site and that the interaction of SR-BI with HVR1 allows for the exposure of CD81-binding region on E2 (54, 60, 61).

Interestingly, most antibodies endowed with broad neutralizing activity target conserved conformational epitopes on E2 and inhibit the interaction between E2 and CD81 (52). Neutralizing antibodies targeting the CD81-binding site either recognize linear epitopes located in amino acids 412–423, conformational epi-topes with contact residues located between residues 523 and 535 or epitopes spanning these two CD81-binding regions (Figure 1). Importantly, the most potent neutralizing murine antibodies target linear epitopes covering residues 412–423, whereas antibodies isolated from HCV-infected patient sera targeting this epitope are rare. By contrast, most human neutralizing antibodies recognize conformational epitopes centered on the CD81-binding residues W529, G530, and D535 (62).

While most neutralizing antibodies target CD81-binding sites, some neutralizing antibodies targeting conserved epitopes overlapping the SR-BI binding site have been described (63). Interestingly, a synergistic neutralization has been observed between the HEPC74 and the HEPC98 antibodies that, respectively, block E2-CD81 and E2-SR-BI binding (64). Moreover, the pairing of these antibodies showed an enhanced neutralizing breadth and their mechanisms of action were found to be independent. Thus, the reliance of HCV on multiple cellular receptors constitutes a source of vulnerability that could be exploited in the design of an efficient vaccine.

Over the past few years, the interaction between E2 and several different neutralizing antibodies could be precisely mapped by resolution of the crystal structure of E2 peptide-antibody complexes (23, 65–67), providing a molecular framework to better understand HCV neutralization.

### Role of Glycosylation in HCV Resistance to Neutralization

The most common mechanism of evasion to antibody neutralization is mutational escape. For its replication, HCV relies on a RNA-dependent RNA polymerase that lacks proofreading capabilities and allows a high replication rate of the virus. These features result in the generation of a high diversity of viral variants that constitute quasispecies (68). The neutralization escape variants contained in the viral population have a selective advantage over sensitive variants and can become the dominant circulating strain (52, 69–71).

Apart from its high genetic heterogeneity, HCV has developed various ways to escape the host immune response. One of them is a protection by a glycan shield that reduces the immunogenicity of the envelope proteins and masks conserved neutralizing epitopes at their surface. Indeed, glycans associated with viral envelope proteins are synthesized by the host cell and are recognized as self-structures. Thus, many viruses that impact human health use glycosylation to evade the host immune response (72).

Characterization of N-glycosylation mutants in the HCVcc system has shown that at least five glycans (E2N1, E2N2, E2N4, E2N6, and E2N11) on E2 reduce the sensitivity of the virus to neutralization (32). Indeed, the absence of one of these glycans leads to an increase in the sensitivity of the virus to neutralization by antibodies purified from HCV positive patients or mAbs. These data further confirm those obtained with the HCVpp system for E2N1, E2N6, and E2N11 mutants (8, 31). However, in this latter system, E2N2 and E2N4 mutations resulted in the production of non-infectious particles. HCV glycans have been shown to mask the neutralizing activity of mAbs targeting conserved epitopes while having no effect on the recognition of HVR1 epitopes (32). Moreover, E2N1, E2N2, E2N4, and E2N6 modulate the inhibition of HCV infectivity by a soluble form of CD81 receptor. Since most broadly neutralizing mAbs target CD81-binding site on E2, these results suggest that this site is the neutralizing antibody target that is protected by N-glycans. In agreement with these data, the modeling of N-linked glycans on E2 core structure confirmed the presence of an extensive glycan shield that masks CD81-binding site (Figure 2) (20). Since HVR1 is also thought to hide CD81-binding site, it would be interesting to determine whether glycans and HVR1 shielding effects are additive.

E1 glycosylation had no effect on the sensitivity of HCVpp to neutralization with purified antibodies from HCV positive patient sera (6). However, this result could be due to the fact that the neutralizing immune response against HCV is dominated by anti-E2 antibodies. Thus, it would be interesting to determine the role of E1 glycans in the protection of HCV from neutralization by using anti-E1 neutralizing mAbs. Unfortunately, the availability of such antibodies remains very limited (73, 74).

### N-Glycosylation Escape Mutants

Further highlighting the importance of N-linked glycosylation in shielding E2 epitopes from recognition by broadly neutralizing antibodies, Pantua and collaborators (75) observed the appearance of escape mutants from AP33 neutralizing mAb that exhibited a glycan shift. Indeed, *in vitro* resistance selection led to the identification of N417S and N417T HCVcc variants that were resistant to broadly neutralizing antibodies targeting the 412–423 E2 epitope. The two variants presented a glycosylation shift from N417 (E2N1) to N415. N415 residue has been shown to be important for the recognition of the 412–423 epitope by AP33 and HCV1 neutralizing antibodies. Moreover, N415 appeared to be buried in the antibody-peptide interface in the crystal structure of 412–423 epitope in complex with several neutralizing antibodies (66, 67, 75). Consequently, attachment of a glycan at N415 and not at N417 would create a steric bulk that would abrogate AP33 and HCV1 binding. These data led to the conclusion that the glycosylation shift from residue N417 to N415 causes HCV resistance to AP33 and HCV1 neutralizing antibodies (75).

Interestingly, this glycosylation shift could also be observed in the absence of neutralizing antibody selection, thus showing that residue 417 is polymorphic (N, S, or T) (76–78). Furthermore, in a minority of genotype 3a isolates, an additional glycosylation site appears in the intragenotypic hypervariable region HVR495, which has been shown to provide some protection against neutralizing antibodies (9). Therefore, as previously described for HIV, glycosylation shift is another mechanism leading to the appearance of HCV resistance to neutralizing antibodies (79). However, as compared to HIV, this glycosylation shift remains very limited in HCV as determined by the analysis of patient sequences (80). This is likely due to additional roles played by most HCV glycans in protein folding and/or in other unidentified functions.

However, while conferring resistance to AP33 and HCV1 neutralizing antibodies and a greater *in vitro* fitness, the glycan shift observed in N417T and N417S led to greater sensitivity to neutralization by other antibodies targeting amino acids 412–423 (76, 77). Notably, HC33.1 antibody, which was isolated from an HCV-infected blood donor, could neutralize HCVcc bearing E2 N417T and N417S adaptive mutations more efficiently than HCVcc wild type (wt) (81). The structure of HC33.1 in complex with E2 412–423 epitope revealed a different conformation of the epitope than the beta-hairpin conformation observed in the epitope-AP33 or HCV1 complexes. In this structure, N415 residue was surface exposed such that its glycosylation would not impair antibody binding. Thus, crystallography studies have highlighted the structural flexibility of E2 412–423 epitope that exhibited three different conformations depending on the matched antibody (23, 65–67, 82). The structural flexibility of this peptide may contribute to reduce its immunogenicity in HCV-infected individuals. Such structural flexibility was also reported for E2 epitope 427–446 that presents different structures when bound to neutralizing or non-neutralizing antibodies (83). Moreover, the crystal structure of E2 core revealed that 60% of all residues are either disordered or in loops implying considerable overall flexibility (20, 21). Hence, as proposed for HIV and influenza viruses, conformational flexibility seems to be an additional mechanism used by HCV to evade humoral immunity (84).

### Modulation of Immunogenicity by N-Glycosylation

Since glycans represent one-third of E1E2 heterodimers molecular weight, they are likely to impact the immunogenicity of the envelope proteins. Several data argue in favor of this hypothesis. For instance, removal of E1N4 glycosylation site improved the anti-E1 humoral immune response (85, 86). In a similar way, mutation of E2N9 glycosylation site improved the immunogenicity of E2 in a DNA-based vaccination approach (87). Indeed, E2N9 mutant elicited higher E2-specific cytotoxic T lymphocytes activities, T lymphocyte proliferation, and expression of IFN gamma producing T cells. More recently, mice vaccination with CpG coupled E1E2 DNA containing mutated E1N2 and E2N3 glycosylation sites induced a higher cellular immune response than wt E1E2. Furthermore, the corresponding serum presented broad neutralizing activity (88). Thus, in this DNA vaccination assay, the naturally poor immunogenicity of E1E2 could be enhanced by deletion of N-glycans combined with the addition of immune activator CpG. Recently, HCV E1E2 heterodimer and a mutant form lacking E2N6 glycosylation site were transiently expressed in an edible crop, lettuce, using *Agrobacterium*. Produced antigens were used for oral vaccination of mice. The follow up of the immune response induced by HCV heterodimers revealed improved immunogenic properties for the N-glycosylation mutant compared to wt E1E2 (89).

Interestingly, the type of glycans associated with HCV envelope glycoprotein E2 can also affect its immunogenicity. Indeed, as compared to its expression in mammalian cells, E2 produced in insect cells exhibits different glycosylation patterns, which also lead to increased immunogenicity, as evidenced by the induction of higher titers of broadly neutralizing antibodies (90).

## Conclusion

In conclusion, recent studies have greatly contributed to increase the knowledge of the mechanisms used by HCV to evade humoral immune response. The high genetic variability of the virus favors the emergence of neutralization escape variants. Furthermore, the envelope proteins associated glycans and the virus-associated lipoproteins protect conserved immunogenic epitopes from neu-tralizing antibodies. Although HCV, HIV, and influenza virus share the common feature of shielding neutralizing epitope with glycans, N-glycosylation sites in HCV E1E2 are far less variable than in HIV and influenza, suggesting a different contribution to HCV immune escape (91). Furthermore, the influence of HCV glycans on anti-HCV immune response makes them essential parameters to take into account in the design of an HCV vaccine based on HCV envelope glycoproteins. Indeed, while the removal of N-glycans seems to improve the envelope proteins immunogenicity, the contribution of these carbohydrates to E1 and E2 folding makes them essential components for the induction of a biologically relevant E1E2-specific antibody response. One needs, therefore, to keep a good balance between these two functions to optimize the design of a vaccine based on HCV envelope glycoproteins.

Finally, answering questions that remain on the role played by N-glycan in modulation of the humoral immune response will facilitate the design of an effective HCV vaccine.

## Author Contributions

ML wrote the manuscript. XH and JD revised the manuscript. XH also constructed the structural model.

## Conflict of Interest Statement

The authors declare that the research was conducted in the absence of any commercial or financial relationships that could be construed as a potential conflict of interest.

## Abbreviations

HCV, hepatitis C virus; HVR1, hypervariable region 1; HCVpp, HCV pseudoparticles; HCVcc, cell culture-derived HCV; ER, endoplasmic reticulum; SR-BI, scavenger receptor BI; mAb, monoclonal antibody; Glc, glucose; Man, mannose; GlcNAc, *N*-acetylglucosamine.
